# Alisol A 24-acetate stimulates lipolysis in 3 T3-L1 adipocytes

**DOI:** 10.1186/s12906-021-03296-0

**Published:** 2021-04-22

**Authors:** Hai-xia Lou, Wen-cheng Fu, Jia-xiang Chen, Tian-tian Li, Ying-ying Jiang, Chun-hui Liu, Wen Zhang

**Affiliations:** 1 School of Life Sciences, East China Normal University, 500 Dongchuan Road, Shanghai, 200241 China; 2 China National Institute of Standardization, 4 Zhichun Road, Beijing, 100191 China

**Keywords:** *Alisma orientale* (Sam.) Juz., Alisol A 24-acetate, Lipolysis, 3 T3-L1 adipocyte, Obesity

## Abstract

**Background:**

Alisol A 24-acetate (AA-24-a), one of the main active triterpenes isolated from the well-known medicinal plant *Alisma orientale* (Sam.) Juz., exhibits multiple biological activities including hypolipidemic activity. However, its effect on lipid metabolism in adipocytes remains unclear. The present study aimed to clarify the effect of AA-24-a on adipocyte lipolysis and to determine its potential mechanism of action using 3 T3-L1 cells.

**Methods:**

We assayed the release of glycerol into culture medium of 3 T3-L1 cells under treatment with AA-24-a. Protein and mRNA expression and phosphorylation levels of the main lipases and kinases involved in lipolysis regulation were determined by quantitative polymerase chain reaction and western blotting. Specific inhibitors of protein kinase A (PKA; H89) and extracellular signal-regulated kinase (ERK; PD98059), which are key enzymes in relevant signaling pathways, were used to examine their roles in AA-24-a-stimulated lipolysis.

**Results:**

AA-24-a significantly stimulated neutral lipolysis in fully differentiated adipocytes. To determine the underlying mechanism, we assessed the changes in mRNA and protein levels of key lipolysis-related genes in the presence or absence of H89 and PD98059. Both inhibitors reduced AA-24-a-induced lipolysis. Moreover, pretreatment with H89 attenuated AA-24-a-induced phosphorylation of hormone-sensitive lipase at Ser660, while pretreatment with PD98059 attenuated AA-24-a-induced downregulation of peroxisome proliferator-activated receptor-γ and perilipin A.

**Conclusions:**

Our results indicate that AA-24-a promoted neutral lipolysis in 3 T3-L1 adipocytes by activating PKA-mediated phosphorylation of hormone-sensitive lipase and ERK- mediated downregulation of expression of perilipin A.

**Supplementary Information:**

The online version contains supplementary material available at 10.1186/s12906-021-03296-0.

## Background

Obesity and being overweight have become major public health issues in both children and adults. Obesity increases the risk of many diseases, including type 2 diabetes mellitus, cardiovascular disease, non-alcoholic fatty liver disease and cancer [1]. Obesity is caused by an imbalance between energy intake and energy expenditure, leading to an abnormal increase in body fat [2]. Adipose tissue is the major repository of energy, which is stored in the form of triglycerides that are deposited in lipid droplets (LDs) within adipocytes during periods of excess energy intake, and released in the form of non-esterified fatty acids (FAs) to supply other organs during fasting [3]. To accommodate lipids, the diameter and volume of human adipocytes can change by 20-fold and several thousand-fold, respectively [4]. Therefore, decreasing excess lipid accumulation, with subsequent attenuation of adipocyte hypertrophy, is important for alleviating obesity. Under fed conditions, LDs store triglycerides mainly in adipose tissues; the hydrolysis of ester bonds between long chain FAs and the glycerol backbone in triacylglycerol is called “lipolysis” [5]. Lipolysis is a catabolic process involved in the breakdown of triglycerides and subsequent release of large amounts of energy [6]. Fine regulation of lipolysis is important for maintaining energy homeostasis, and dysregulation of lipolysis can lead to metabolic abnormalities. Reduced lipolysis contributes to obesity through the accumulation of triglycerides in adipose tissue [6, 7]. But excessive lipolysis can increase circulating FA levels and ectopic triglyceride storage, which are associated with detrimental metabolic abnormalities [7]. Therefore, strategies to increase lipolysis may contribute to the alleviation of obesity, if FA utilization increase in response to increased lipolysis [7].

Adipocyte lipolysis proceeds in an orderly and regulated manner, with different enzymes acting at each step [7]. Neutral hydrolysis of triglycerides to FAs and glycerol requires three consecutive steps that involve at least three different enzymes [5]. Adipose triglyceride lipase (ATGL) selectively catalyzes the first step of triglyceride hydrolysis to generate diacylglycerols (DAGs) and free FAs. The second step of lipolysis is dependent on the activation of hormone sensitive lipase (HSL), a multifunctional enzyme that is capable of hydrolyzing both the first and the second step of lipolysis. HSL hydrolyzes DAGs and produces monoacylglycerol (MAG) and FAs. Within the lipolysis cascade, HSL functions as a rate-limiting enzyme for DAG catabolism. In the last step of lipolysis, MAGs are released into the cytosol and eventually cleaved by monoglyceride lipase (MGL) to generate glycerol and FA [5]. ATGL and HSL are quantitatively the most important lipases [8]. HSL is a major target for protein kinase A (PKA)-catalyzed phosphorylation. Other kinases, including adenosine 5′-monophosphate-activated protein kinase (AMPK), extracellular signal-regulated kinase (ERK), glycogensynthase kinase-4, and Ca^2+^/calmodulin-dependent kinase, also phosphorylate HSL to modulate its enzyme activity [9]. Besides lipolytic enzymes, perilipin A, an LD-associated protein, plays an essential role in adipocyte lipolysis [3, 10]. Perilipin A coats LDs and limits access of lipase to the LD, thereby preventing lipolysis under basal conditions [8, 9]. Therefore, decreased protein expression may impair the barrier function of perilipin A and subsequently lead to an increase in lipolysis. During this process, ERK activation is an early signal for the reduction in perilipin protein expression and subsequent induction of lipolysis [11].

*Alisma orientale* (Sam.) Juz. is a well-known medicinal plant from the Alismataceae family that is mainly distributed in China, Russia, Japan, Mongolia, and North India [12]. Rhizoma Alismatis (RA), the dried rhizome of *A. orientale*, is widely used in traditional Chinese medicine (TCM) and is popularly known as Ze Xie in Chinese [12, 13]. Pharmacological studies have revealed various benefits of RA, including diuretic, anti-inflammatory, antitumor, antibacterial, antiviral, anti-diabetic, hepatoprotective, hypolipidemic [12, 13], and anti-obesity [14] activities. Choi et al. reviewed that administration of RA extract markedly decreased body weight and fat mass (abdominal subcutaneous, perirenal, and epididymal fat) in animals [14]. Moreover, RA extract markedly decreased not only fat mass, but also adipocyte size in fat tissue [14], indicating its potential for triglyceride breakdown. However, the active compound in RA that exerts this activity is unclear. Alisol A 24-acetate (AA-24-a), one of the main active triterpenoid compounds isolated from RA, has been used as a marker for quality control of the crude drug [15]. AA-24-a was previously shown to decrease serum cholesterol and triglyceride levels in animal models [16, 17], and to suppress triglyceride accumulation in HepG2 cells [18]. However, whether AA-24-a has an active inhibitory effect on triglyceride accumulation in adipocytes remains unknown. Moreover, knowledge regarding its effects on triglyceride metabolism, especially lipolysis, in adipocytes is relatively limited.

Therefore, the present study aimed to elucidate the effect of AA-24-a on lipolysis in 3 T3-L1 adipocytes and to determine its potential mechanism of action. We hypothesized that AA-24-a decreases the amount of intracellular triglycerides in adipocytes by promoting lipolysis. To test this hypothesis, fully differentiated adipocytes were treated with AA-24-a and the resulting effects on lipolysis, lipolytic enzymes, and pathways involved in adipocyte lipolysis were investigated.

## Methods

### Materials

**Fig. 1:**
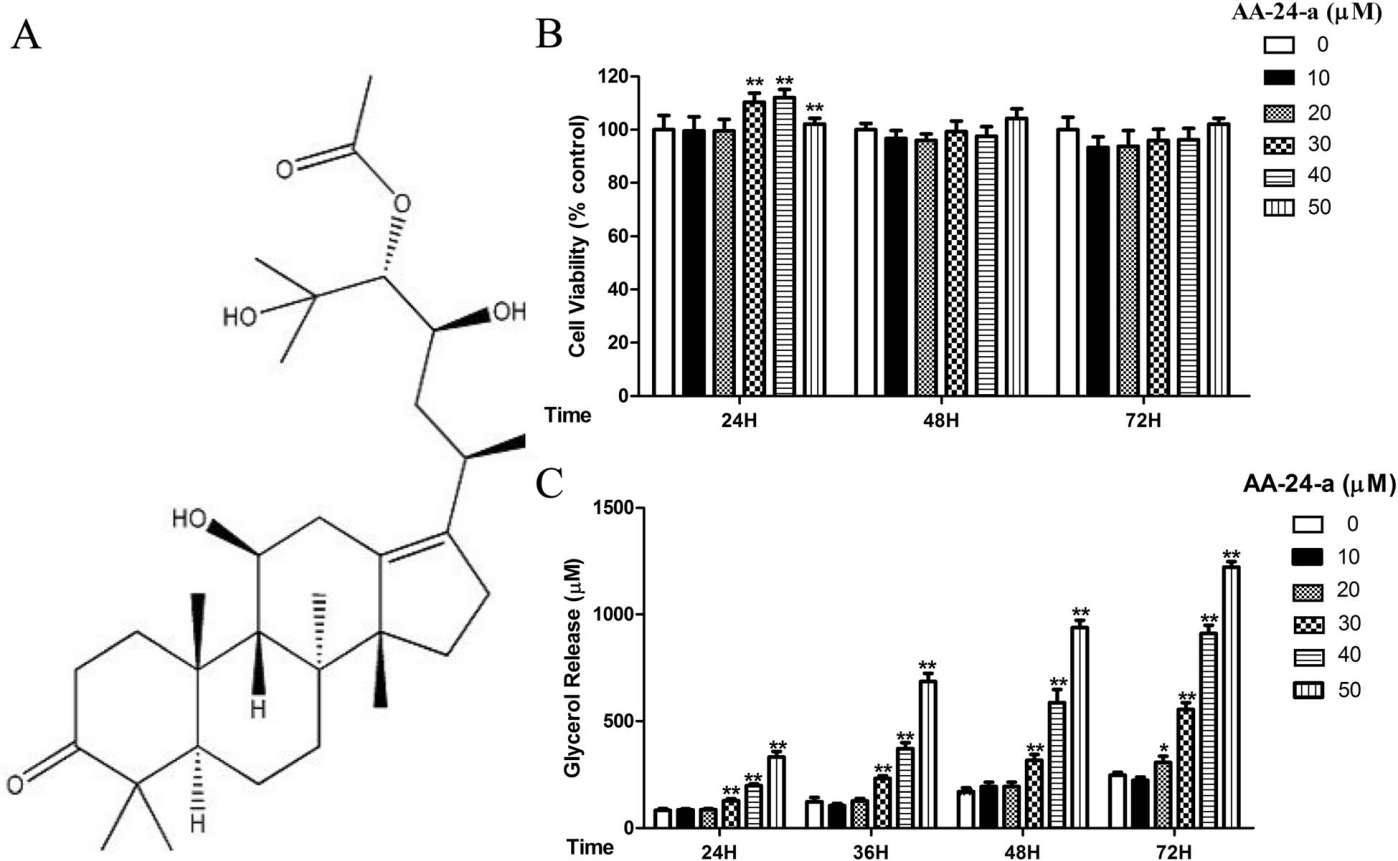
Effects of AA-24-a on lipolysis and triglyceride accumulation in 3 T3-L1 adipocytes. **a** Chemical structure of AA-24-a. **b** Cell viability as determined by the MTT assay (*n* = 6). **c** Amounts of glycerol released into the culture medium measured using a glycerol assay kit (*n* = 4). Data are presented as mean ± standard deviation. *P < 0.05, **P < 0.01, versus control cells. OD: optical density

AA-24-a (structure shown in Fig. 1a) extracted from RA (purity: 98.81% by high-performance liquid chromatography) was obtained from Chengdu Herbpurify (Chengdu, China). Dulbecco’s modified Eagle’s medium (DMEM), penicillin/streptomycin, and newborn calf serum (NCS) were obtained from Gibco (Gaithersburg, MD, USA). Fetal bovine serum (FBS) was purchased from Bovogen Biologicals (East Keilor, VIC, Australia). Methyl thiazolyl tetrazolium (MTT), dimethyl sulfoxide (DMSO), Oil Red O, and dexamethasone were purchased from Sigma-Aldrich (St. Louis, MO, USA). Indomethacin and 3-isobutyl-1-methylxanthine (IBMX) were obtained from Aladdin Inc. (Shanghai, China). FA-free bovine serum albumin (BSA), insulin (bovine), and Alexa Fluor 680 AffiniPure Goat Anti-Rabbit IgG (H + L) were purchased from Yeasen Biotechnology (Shanghai, China). Isoprenaline (ISO) was obtained from Dalian Meilun Biotechnology (Dalian, China). H89, PD98059, and Compound C were obtained from Selleck Chemicals (Houston, TX, USA). Antibodies against HSL, phospho-HSL (Ser563), phospho-HSL (Ser565) and phospho-HSL (Ser660) were obtained from Cell Signaling Technology (Danvers, MA, USA). Antibodies against β-actin, peroxisome proliferator-activated receptor (PPAR)-γ, and extracellular signal-regulated kinase (ERK) 1/2 were purchased from Proteintech (Rosemont, IL, USA). Antibodies against phospho- AMPK (Thr183/Thr172) and phospho-ERK (Thr202/Tyr204) were purchased from Abgent (San Diego, CA, USA).

### Cell culture and differentiation

Three T3-L1 mouse fibroblast cells were obtained from the National Center for Drug Screening (Shanghai, China). Differentiation was induced as previously described [19]. Briefly, 3 T3-L1 preadipocytes were cultured in DMEM containing 10% NCS, 100 U/mL penicillin, and 100 μg/mL streptomycin at 37 °C under 5% CO_2_. For adipocyte differentiation, 2-day post-confluent 3 T3-L1 cells were exposed to differentiation medium (DMEM containing 10% FBS, 10 μg/mL insulin, 0.5 μM IBMX, 1 μM dexamethasone, and 0.2 mM indomethacin) for 3 days. The culture medium was then changed to DMEM supplemented with 10% FBS and 10 μg/mL insulin for a further 3 days. Subsequently, the cells were maintained in DMEM containing 10% FBS for an additional 2 days. Afterward, the fully differentiated adipocytes were harvested for further experiments.

### MTT assay

The 3 T3-L1 fibroblast cells were seeded into 96-well plates at a density of 1 × 10^4^ cells/well. After differentiation into mature adipocytes, the cells were incubated in DMEM containing 0.2% BSA for 12 h. The culture medium was then changed to DMEM containing 0.2% BSA and 10–50 μM AA-24-a, and the cells were incubated for 24, 48, or 72 h. Following the incubation, 20 μL of 3 mg/mL MTT was added to the culture medium. After 3 h, the resulting insoluble formazan crystals were dissolved by adding DMSO (200 μL/well), and the absorbance was measured at 490 nm. Cell viability was calculated as follows:

$$ Cell\ viability\%=\frac{\mathrm{ODsample}-\mathrm{ODblank}}{\mathrm{ODcontrol}-\mathrm{ODblank}}\times 100\% $$

where OD is the optical density at 490 nm.

### Lipolysis measurement

Lipolysis was evaluated by measuring the amount of glycerol released into the culture medium. After serum starvation, differentiated 3 T3-L1 adipocytes were treated with 10–50 μM AA-24-a for 24, 36, 48, or 72 h. After the treatment, the cell culture supernatants were collected from the wells and the released glycerol was measured using a glycerol assay kit (Applygen, Beijing, China).

### Gene expression analysis

Total RNA was extracted from AA-24-a-treated 3 T3-L1 cells using TRIzol reagent (Takara, Kusatsu, Japan) in accordance with the manufacturer’s protocol. Extracted RNA was dissolved in diethyl pyrocarbonate-treated water, quantified by the ratio of absorbance at 260 and 280 nm, and sample integrity was verified by 1% agarose gel electrophoresis. Complementary DNA (cDNA) was synthesized from 1 μg total RNA in 20 μL using a PrimeScript™ RT Reagent Kit (Perfect Real Time) and random primers (Takara, Kusatsu, Japan). The cDNA was subjected to real-time quantitative polymerase chain reaction (qPCR) using an UltraSYBR Mixture Kit (Cwbio, Beijing, China) and the CFX96TM Real-Time PCR Detection System (Bio-Rad, Hercules, CA, USA). Reactions were performed in a 25 μL mixture comprising 12.5 μL of 2 × SYBR Green reaction buffer, 1 μL cDNA, and 200 nM of each primer. The thermal cycling conditions were as follows: initial denaturation at 95 °C for 10 min, followed by 40 cycles of 95 °C for 15 s and 60 °C for 1 min. The sequences of the primers used for real-time qPCR were listed in Table 1, and all target mRNA levels were normalized to β-actin mRNA levels in each well as an internal standard. Fold expression was defined as the fold increase relative to controls.

**Table 1:** Sequences of primers used for real-time qPCR

Gene name	Forword primer (5′-3′)	Reverse primer (5′-3′)
β-actin	CGCTCGTTGCCAATAGTG	GCTGTGCTATGTTGCTCTAG
MGL	CGGACTTCCAAGTTTTTGTCAGA	GCAGCCACTAGGATGGAGATG
HSL	AGACCACATCGCCCACA	CCTTTATTGTCAGCTTCTTCAAGG
ATGL	GACCTGATGACCACCCTTTC	TGTTTGGCTTTATCTCGGCTC
PPARγ	CAAGAATACCAAAGTGCGATCAA	GAGCTGGGTCTTTTCAGAATAATAAG
Perilipin A	GCTCTTCAATACCCTCCAGAAAAG	TTCGAAGGCGGGTAGAGATG
PGC-1α	CCCTGCCATTGTTAAGACC	TGCTGCTGTTCCTGTTTTC
UCP-1	CCTGCCTCTCTCGGAAACAA	GTAGCGGGGTTTGATCCCAT
PPARα	AACATCGAGTGTCGAATATGTGG	CCGAATAGTTCGCCGAAAGAA

### Western blotting

To extract protein, cells were washed twice with ice-cold PBS and harvested in radioimmunoprecipitation assay lysis buffer (150 mM sodium chloride, 1.0% Triton X-100, 0.5% sodium deoxycholate, 0.1% sodium dodecyl sulfate [SDS], 50 mM Tris pH 8.0) containing protease and phosphatase inhibitors. Cell lysates were incubated on ice for 30 min, vortexing every 5 min, then stored at − 80 °C overnight; samples were thawed on ice and then spun by centrifugation at 12,000 rpm at 4 °C for 20 min. Protein concentrations were determined with a Bicinchoninic Acid Protein Assay Kit (Yeasen Biotechnology, Shanghai, China) according to the manufacturer’s protocol. Equal amounts of protein (40 μg) were separated by 12% SDS-polyacrylamide gel electrophoresis and transferred onto nitrocellulose membranes. After blocking in 5% non-fat dried milk solution for 1 h at room temperature, the membranes were incubated overnight at 4 °C with primary antibodies diluted in Tris-buffered saline containing 0.1% Tween-20 (TBST). After three washes in TBST, the membranes were incubated for 1 h with secondary antibodies at room temperature. The antibody-bound protein bands were washed and then visualized on an Odyssey CLx Imaging System (LI-COR, Lincoln, NE, USA), and all blots were analyzed by Image-Pro Plus software (Media Cybernetics, Rockville, MD, USA).

### Statistical analysis

Data are expressed as mean ± standard deviation. Statistically significant differences among experimental groups were determined by one-way analysis of variance followed by Duncan or Dunnett’s multiple-comparisons tests using SPSS software (IBM, Armonk, NY, USA). *P* values of < 0.05 and < 0.01 were considered to indicate significant and extremely significant differences, respectively.

## Results

### AA 24-a stimulates basal lipolysis in 3 T3-L1 adipocytes

The potential cytotoxicity of AA-24-a toward fully differentiated 3 T3-L1 adipocytes was evaluated by the MTT assay. As shown in Fig. 1b, treatment with 30, 40, or 50 μM AA-24-a for 24 h increased cell viability relative to untreated control cells by 10.3, 12.0, and 10.8%, respectively. Treatment for 48 or 72 h had no effect on cell viability.

To determine whether AA-24-a possesses lipolytic activity, the amount of glycerol released into the culture medium was measured as an index of lipolysis. As shown in Fig. 1c, 10 μM AA-24-a had no effect compared with untreated control cells at any time point, whereas 20 μM AA-24-a facilitated a significant increase in the release of glycerol from adipocytes at 72 h (*P* < 0.05). Treatment with higher concentrations of AA-24-a (30, 40, or 50 μM) showed significant increases in glycerol release that started at 24 h and increased over time (*P* < 0.01). Treatment with 50 μM AA-24-a for 24, 36, 48, or 72 h increased glycerol release by 297.2, 452.8, 452.5, and 394.4% relative to control cells, respectively. These findings revealed that AA-24-a significantly induced basal lipolysis in fully differentiated 3 T3-L1 adipocytes.

### AA 24-a affects lipolytic enzymes and key proteins involved in the ERK pathway

**Fig. 2:**
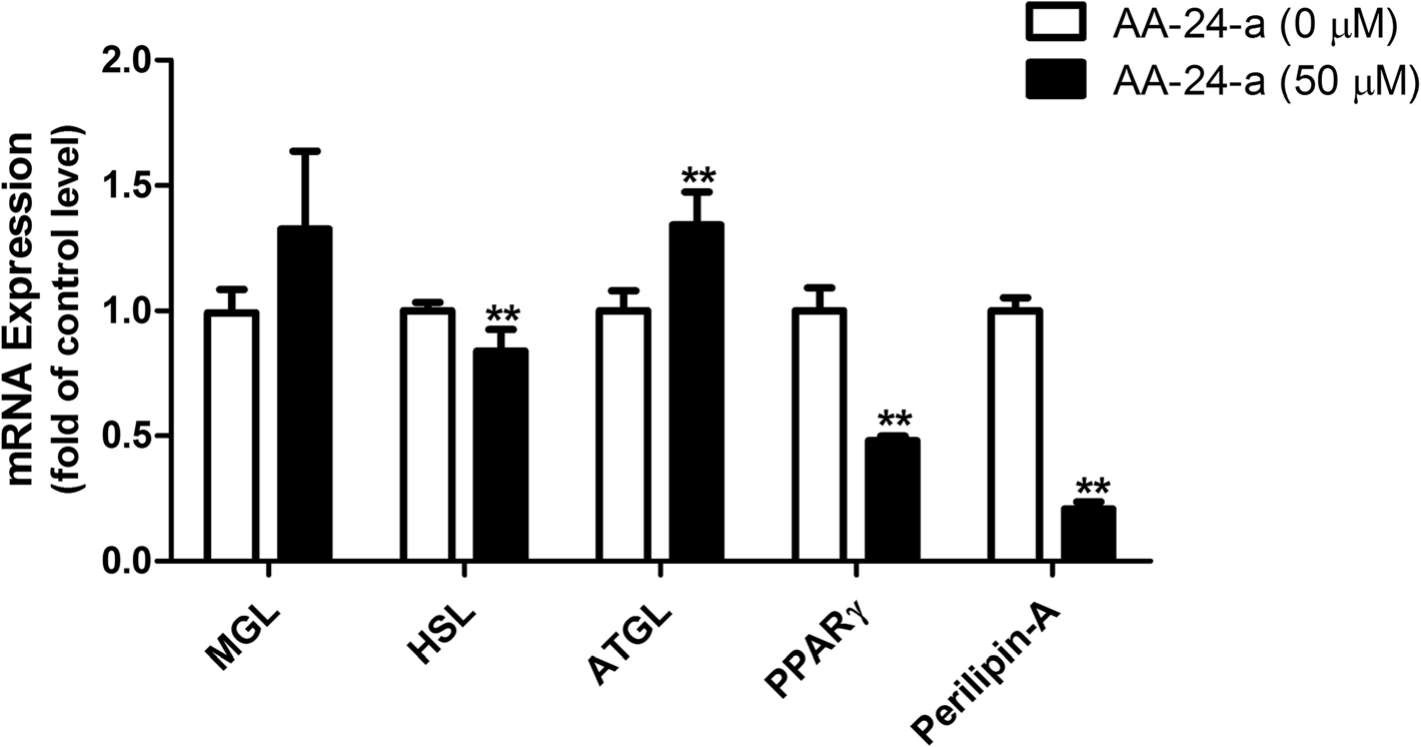
Relative levels of mRNA expression of lipolysis-related genes in 3 T3-L1 adipocytes in response to 12 h of treatment with AA-24-a as measured by qPCR. **P < 0.01 versus control cells

The effects of AA-24-a on mRNA expression of five lipolysis-related genes were evaluated using qPCR. As shown in Fig. 2, treatment 3 T3-L1 cells with 50 μM AA-24-a for 12 h resulted in a significant decrease in mRNA expression of HSL, PPARγ and perilipin A (*P* < 0.01), and a significant increase in ATGL expression (*P* < 0.01).

**Fig. 3:**
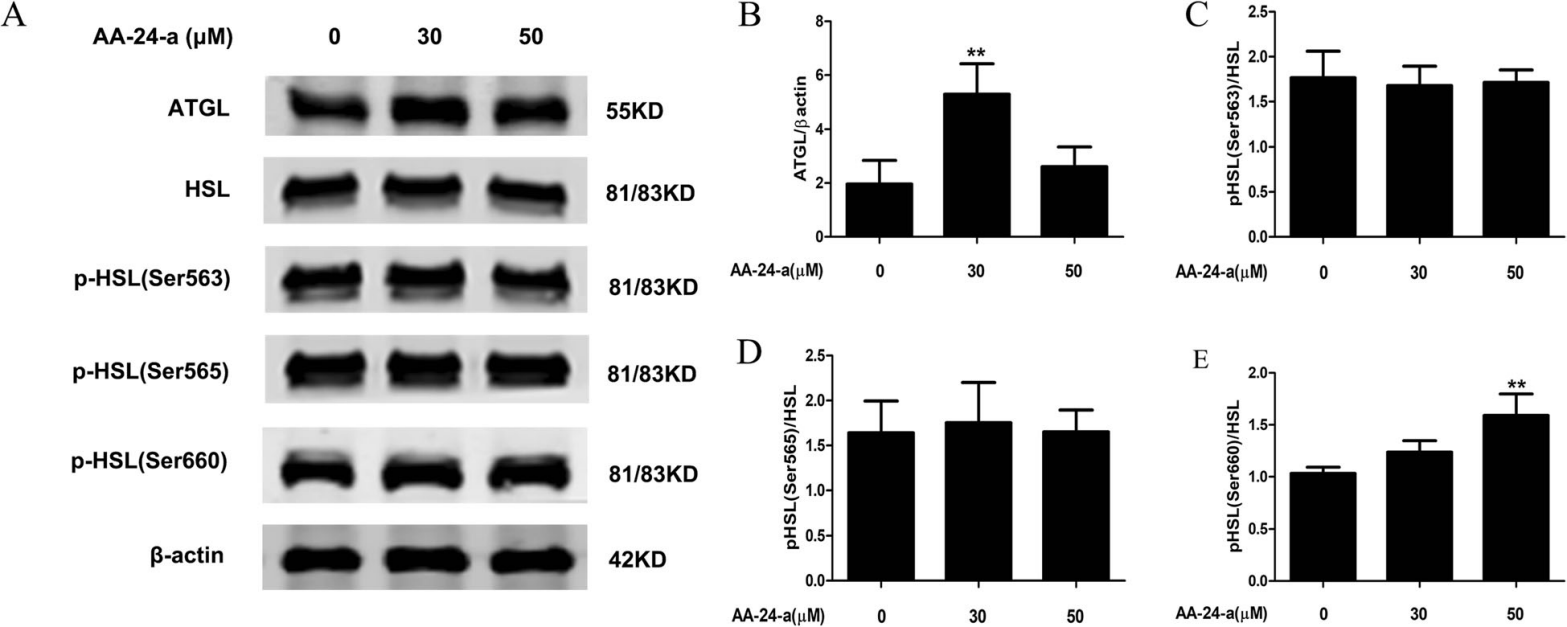
Effects of AA-24-a on phosphorylation of HSL and expression of ATGL in 3 T3-L1 adipocytes. **a** Western blotting of ATGL, HSL, and phospho-HSL (Ser563, Ser565, and Ser660) after treatment with AA-24-a (0, 30 or 50 μM) for 36 h. **b–e** Quantification of protein band densities. Data are presented as mean ± standard deviation (*n* = 3 or 4). **P < 0.01, versus control cells

Neutral lipolysis in adipocytes is mediated by lipolytic enzymes. ATGL and HSL are quantitatively the most important lipases [8], and the lipolytic activity of HSL is dependent on phosphorylation [20]. Thus, to determine the underlying molecular mechanism by which AA-24-a stimulates adipocyte lipolysis, we investigated its effects on ATGL protein expression and HSL phosphorylation. Western blotting analysis revealed that 30 μM AA-24-a significantly promoted ATGL protein expression after 36 h of treatment (*P* < 0.01, Fig. 3a and b). While AA-24-a had no effect on HSL phosphorylation at Ser563 or Ser565 (*P* > 0.05; Fig. 3a, c, and d), HSL phosphorylation at Ser660 was enhanced in a dose-dependent manner (Fig. 3a and e). Treatment with 50 μM AA-24-a significantly increased HSL phosphorylation at Ser660 by 54.2% compared with control cells (*P* < 0.01), suggesting that the induced lipolytic effect of AA-24-a involved the PKA pathway. In contrast, AA-24-a treatment had no effect on AMPK-mediated phosphorylation of HSL at Ser565.

**Fig. 4:**
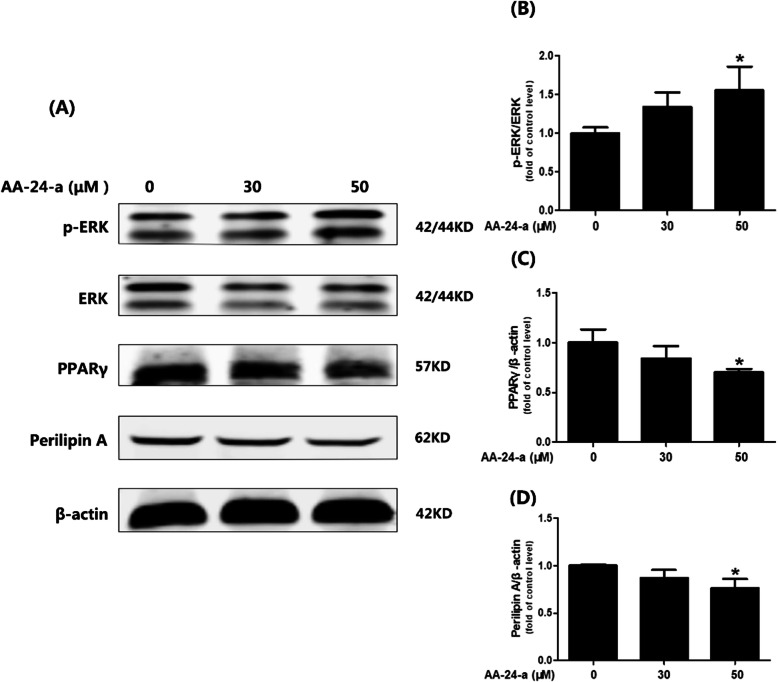
Effects of AA-24-a on phosphorylation of ERK, and expression of PPARγ and perilipin A in 3 T3-L1 adipocytes. **a** Western blotting of phospho-ERK, PPARγ and perilipin A after treatment with AA-24-a (0, 30 or 50 μM) for 24 h. **b–d** Quantification of protein band densities. Data are presented as mean ± standard deviation (*n* = 3). *P < 0.05, versus control cells

The ERK pathway is involved in the regulation of lipolysis [21]. Therefore, we also investigated the effects of AA-24-a on the ERK pathway. We found that treatment with 50 μM AA-24-a significantly increased ERK phosphorylation by 55.6% compared with control cells (*P* < 0.05, Fig. 4a and b). ERK activation can directly reduce perilipin levels through PPARγ, thereby increasing lipolysis [11, 22]. We observed that treatment with 50 μM AA-24-a caused significant downregulation of PPARγ (Fig. 4a and c) and perilipin A (Fig. 4a and d) protein levels compared with control cells (*P* < 0.05).

### AA 24-a increases phosphorylation of HSL by activating the PKA pathway

**Fig. 5:**
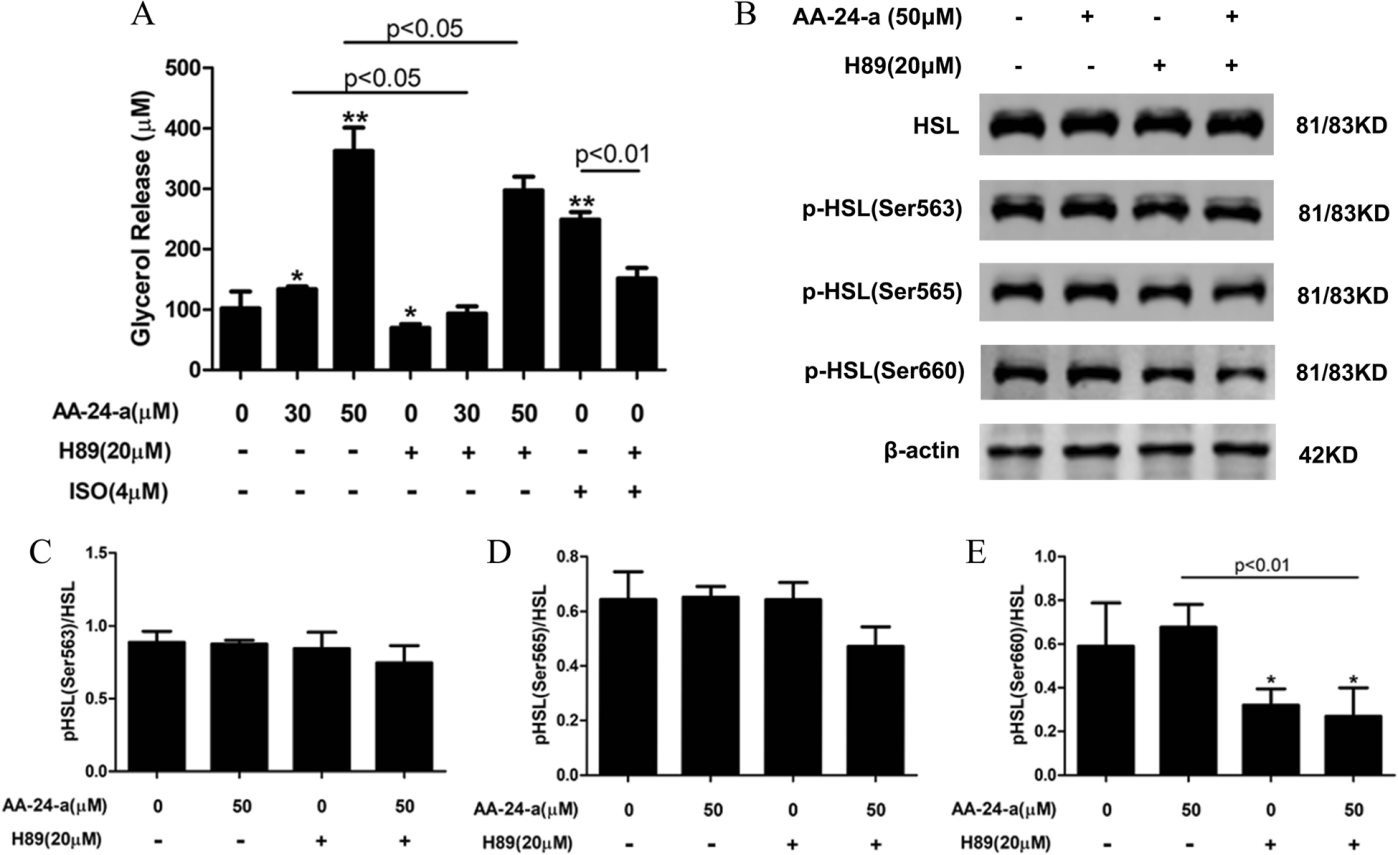
Effects of the PKA inhibitor H89 on the induced lipolytic effect of AA-24-a in 3 T3-L1 adipocytes. **a** Glycerol release into the culture medium. **b** Western blotting of HSL and phospho-HSL (Ser563, Ser565, and Ser660) in adipocytes preincubated with 20 μM H89 for 1 h followed by treatment with 50 μM AA-24-a for 24 h. **c–e** Quantification of protein band densities. Data are presented as mean ± standard deviation (*n* = 3 or 4). *P < 0.05, **P < 0.01, versus control cells. ISO: isoprenaline

To characterize the involvement of the PKA signaling pathway, we examined the effect of the PKA inhibitor H89 on lipolysis induced by AA-24-a. As shown in Fig. 5a, pretreatment of 3 T3-L1 adipocytes with H89 significantly inhibited the lipolysis induced by treatment with 30 or 50 μM AA-24-a for 24 h (*P* < 0.05 and *P* < 0.01, respectively). Inhibition of PKA with H89 also suppressed the 50 μM AA-24-a-induced phosphorylation of HSL at Ser660 by 45.7% compared with AA-24-a treatment alone (*P* < 0.01, Fig. 5b and e). These results indicate that the induced lipolytic effect of AA-24-a was partly mediated by PKA-induced phosphorylation of HSL at Ser660.

### AA 24-a downregulates perilipin expression through ERK activation

**Fig. 6:**
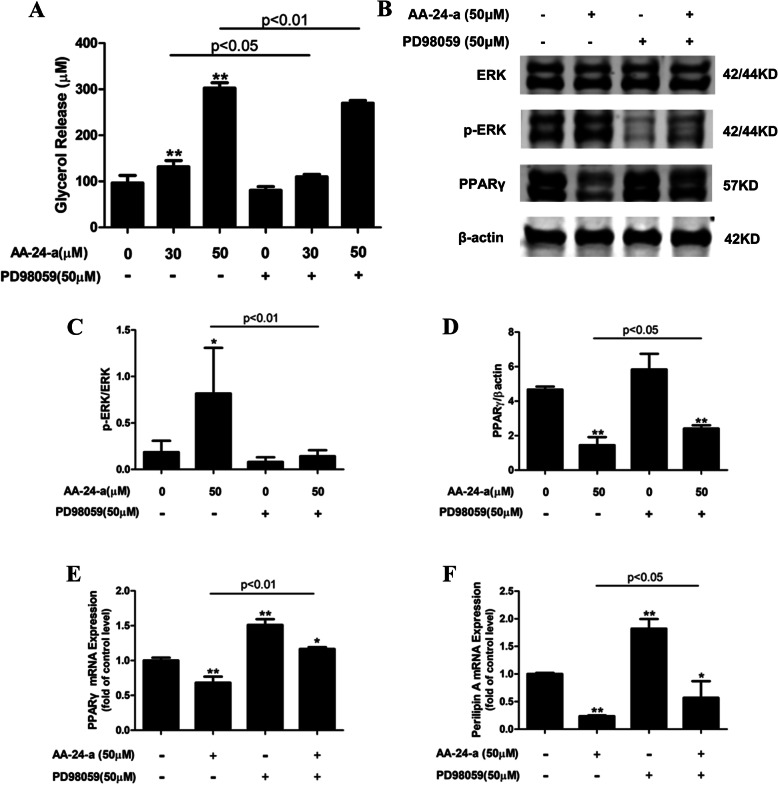
Effects of the ERK inhibitor PD98059 on the induced lipolytic effect of AA-24-a in 3 T3-L1 adipocytes. **a** Glycerol release into the culture medium. **b** Western blotting of ERK, phospho-ERK, and PPARγ in adipocytes preincubated with 50 μM PD98059 for 30 min followed by treatment with AA-24-a for 20 h. **c, d** Quantification of protein band densities. **e, f** mRNA expression levels of PPARγ and perilipin A determined using qPCR. Data are presented as mean ± standard deviation (*n* = 3 or 4). *P < 0.05, **P < 0.01, versus control cells

To further investigate the role of the ERK pathway in AA-24-a-induced lipolysis, the ERK inhibitor PD98059 was employed to examined its effect on lipolysis induced by AA-24-a. First, we evaluated the effects of PD98059 on AA-24-a-induced adipocyte lipolysis. Preincubation of adipocytes with 50 μM PD98059 for 30 min, followed by treatment with 30 or 50 μM AA-24-a for 20 h in the presence of PD98059 significantly reduced the amount of glycerol released into the culture medium compared with AA-24-a treatment alone (*P* < 0.05; *P* < 0.01, respectively; Fig. 6a). Moreover, pretreatment with PD98059 decreased the AA-24-a-stimulated ERK phosphorylation by 82.6% (*P* < 0.01 versus 50 μM AA-24-a treatment alone; Fig. 6b and c). Pretreatment with PD98059 also reversed the AA-24-a-induced decrease in PPARγ, leading to a 24.9% increase in its protein level (*P* < 0.05 versus AA-24-a treatment alone; Fig. 6b and d). Because PPARγ is a transcription factor, we investigated the effects of PD98059 on AA-24-a-mediated transcriptional regulation of PPARγ and its target, perilipin A. Treatment with AA-24-a significantly inhibited mRNA expression of PPARγ (*P* < 0.01; Fig. 6e) and perilipin A (*P* < 0.01; Fig. 6f). Compared with AA-24-a treatment alone, PD98059 treatment reversed this inhibition on PPARγ (*P* < 0.01; Fig. 6e) and perilipin A (*P* < 0.05; Fig. 6f). These results indicate that the induced lipolytic effect of AA-24-a was mediated through activation of the ERK pathway, and that AA-24-a-induced inhibition of PPARγ and perilipin A expression was dependent on the ERK pathway.

## Discussion

The present study demonstrated lipolytic activity of AA-24-a in 3 T3-L1 adipocytes. We showed that AA-24-a stimulated adipocyte lipolysis and subsequently inhibited intracellular lipid accumulation without affecting cell viability.

Lipolysis is a biochemical catabolic pathway that relies on direct activation of the LD-associated lipases ATGL, HSL, and MGL [5]. In the present study, AA-24-a significantly increased the mRNA and protein levels of ATGL, the main triacylglycerol lipase in adipose tissue. ATGL hydrolyzes triacylglycerols to diacylglycerols as the first step of lipid hydrolysis, thereby providing a substrate for HSL in the lipolytic cascade [23].

The main diacylglycerol hydrolase is HSL, which predominantly cleaves FA residues in the sn-1 or sn-3 position of diacylglycerols [24]. HSL exhibits much broader substrate specificity than ATGL and can hydrolyze ester bonds in triacylglycerols, diacylglycerols, monoacylglycerols, cholesteryl esters, retinyl esters, and short-chain carbonic acid esters [24]. The lipolytic activity of HSL is dependent on phosphorylation [25]. PKA is the major kinase involved in phosphorylation of HSL at Ser563, Ser659, and Ser660, and these post-translational modifications lead to HSL activation. Phosphorylation of HSL at Ser565, which is carried out by AMPK, blocks HSL activation to decrease its activity [8]. In the present study, we observed that treatment with AA-24-a decreased the mRNA expression of HSL, but selectively induced HSL phosphorylation at Ser660, with no effect on Ser563 or Ser565. Even though HSL mRNA expression was decreased, the lipolytic activity of HSL is dependent on its phosphorylation, with phosphorylation of HSL leading to increased lipolysis. Previous studies also reported that lipolysis can occur concomitantly with decreased HSL mRNA expression [22, 26]. Phosphorylation at Ser659 and Ser660 has been reportedly critical for controlling HSL activity [25]. Based on these results, we hypothesized that AA-24-a stimulates lipolysis by activating the PKA pathway, resulting in phosphorylation of HSL. Therefore, we used H89, a specific PKA inhibitor, to test this hypothesis. We found that H89 significantly attenuated the AA-24-a-stimulated release of glycerol and phosphorylation of HSL at Ser660, indicating that AA-24-a-mediated lipolysis is partly dependent on activation of the PKA signaling pathway.

The ERK pathway is involved in the regulation of lipolysis through a number of mechanisms [21]. ERK directly phosphorylates HSL at Ser600 to enhance its activity [27]. Moreover, ERK activation can directly reduce perilipin levels through PPARγ, thereby increasing lipolysis [11, 22]. Perilipin is a target gene for PPARγ [28–30]. Activation of ERK downregulates PPARγ transcriptional activity, leading to a decrease in the transcription of perilipin through its PPAR response element and consequent promotion of lipolysis in adipocytes [22]. In the present study, incubation with AA-24-a significantly increased lipolysis, which was accompanied by increased ERK phosphorylation and decreased expression of PPARγ and perilipin A in mRNA and protein level. We speculated that downregulation of perilipin A expression is one of the mechanisms by which AA-24-a induces lipolysis, and that the ERK pathway is involved in the regulation of perilipin A expression. We used the ERK inhibitor PD98059 to test this hypothesis and found that it partially blocked AA-24-a-induced lipolysis. Meanwhile, the AA-24-a-induced downregulation of PPARγ and perilipin A expression was reversed in the presence of PD98059. These results indicated that AA-24-a-induced lipolysis was dependent on the downregulation of perilipin A expression via ERK activation. Moreover, AA-24-a also suppressed PPARγ expression through ERK activation. Perilipin is transcriptionally regulated by PPARγ [22]. The ERK-dependent downregulation of PPARγ and perilipin A expression observed in the present study suggests that AA-24-a probably downregulates perilipin A via an ERK-mediated decrease in PPARγ expression. However, further studies are necessary to confirm this speculation.

In this study, we observed that the inhibitory effects of H89 and PD98059 on AA-24-a-induced lipolysis were partial, with some residual lipolysis. This suggested the involvement of other pathways in AA-24-a-stimulated triglyceride breakdown. In addition to lipolysis by cytosolic lipases, autophagy has been recognized as a complementary pathway for cellular lipid breakdown. The selective breakdown of LD-stored lipids by autophagy, termed lipophagy, is a lysosomal lipolytic pathway that complements the actions of cytosolic neutral lipases [31]. Cellular lipid breakdown from LD stores depends on the direct actions of cytosolic neutral lipases on LDs, as well as the actions of acidic lysosomal lipases on LDs delivered to lysosomes by macroautophagy [31].

In recent studies, induction of autophagy by AA-24-a has been observed in human renal proximal tubular cells, human hepatic stellate cells, and a nonalcoholic steatohepatitis mouse model [32, 33]. AA-24-a was shown to ameliorate nonalcoholic steatohepatitis by inhibiting oxidative stress and stimulating autophagy in both mouse model and human hepatic stellate cells, and to stimulate autophagy via the AMPK/mTOR/ULK1 pathway [33]. Therefore, it is possible that AA-24-a breaks down cellular lipids in adipocytes by activating autophagy. However, further studies are necessary to investigate this possibility.

Strategies aimed at increasing lipolysis may be useful for preventing obesity. But excessive lipolysis can increase circulating FA levels and ectopic triglyceride storage, which are associated with detrimental metabolic consequences such as insulin resistance [7]. Therefore, this option may only be considered if it is associated with oxidation of the newly released fatty acid. Previous findings have demonstrated that adipocytes can increase FA utilization in response to increased lipolysis [7, 34]. Moreover, adipocyte lipolysis does not activate inflammatory pathways in adipose tissue macrophages. Instead, macrophages scavenge excess FAs and convert them into triglycerides stored within multilocular LDs [35]. Therefore, one approach to the prevention and treatment of obesity consists in molecules stimulating lipolysis and oxidation of the released FAs [36]. In the present study, essential factors involved in FA oxidation, AMPK, PPARα, and PPARγ coactivator-1α were activated in AA-24-a-treated cells compared with untreated cells (*P* < 0.01 for all; Supplementary Figs. S1 and S2), suggesting that AA-24-a probably stimulated FA oxidation.

## Conclusions

**Fig. 7:**
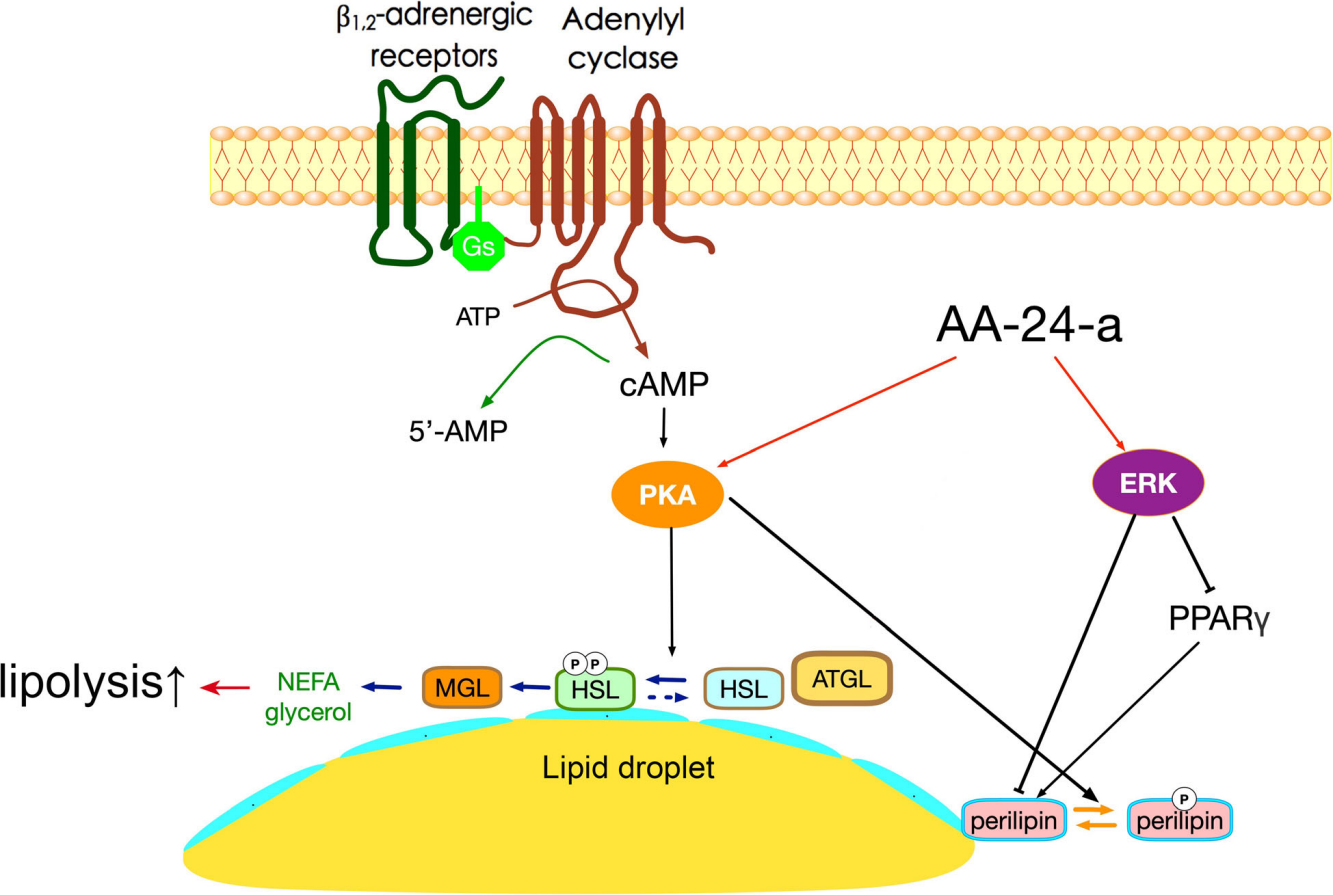
Potential mechanisms by which AA-24-a stimulated lipolysis in 3 T3-L1 adipocytes

The present study demonstrated that AA-24-a induced HSL phosphorylation and suppressed mRNA and protein levels of perilipin A in fully differentiated 3 T3-L1 adipocytes, thereby increasing lipolysis. The AA-24-a-induced lipolysis was mediated by at least two pathways: the first involved PKA activation leading to phosphorylation of HSL at Ser660, and the second involved AA-24-a-induced phosphorylation of ERK leading to decreased expression of PPARγ and perilipin A (summarized in Fig. 7). Our results highlight the potential of AA-24-a as a promising therapeutic agent for obesity and obesity-related disorders.

## Supplementary Information

**Additional file 1: Supplementary Fig. S1.** Relative mRNA expression levels of fatty acid oxidation-related genes in 3 T3-L1 adipocytes in response to treatment with AA-24-a for 12 h as measured by qPCR. Data are presented as mean ± standard deviation (*n* = 6). **P < 0.01, versus control cells. PGC-1α: PPARγ coactivator-1α; UCP-1: uncoupling protein-1.

**Additional file 2: Supplementary Fig. S2.** Effects of AA-24-a on AMPK and phospho-AMPK in 3 T3-L1 adipocytes. (A) Western blot analysis of protein expression after treatment with 0, 30, or 50 μM AA-24-a for 36 h. (B) Quantification of protein band densities as relative values against total β-actin. Data are presented as mean ± standard deviation (*n* = 3 or 4). *P < 0.05, **P < 0.01, versus control cells.

**Additional file 3: Supplementary Material.** Full western blots of proteins.

## Data Availability

The datasets used and/or analyzed during this study are available from the corresponding author on reasonable request.

## Abbreviations

AA-24-a: Alisol A 24-acetate
AMPK: Adenosine 5′-monophosphate-activated protein kinase
ATGL: Adipose triglyceride lipase
BSA: Bovine serum albumin
DMEM: Dulbecco’s modified Eagle’s medium
DMSO: Dimethyl sulfoxide
ERK: Extracellular signal-regulated kinase
FA: Fatty acid
FBS: Fetal bovine serum
HSL: Hormone-sensitive lipase
IBMX: 3-isobutyl-1-methylxanthine
ISO: Isoprenaline
LD: Lipid droplet
MGL: Monoglyceride lipase
MTT: Methyl thiazolyl tetrazolium
NCS: Newborn calf serum
PBS: Phosphate-buffered saline
PKA: Protein kinase A
PPAR: Peroxisome proliferator-activated receptor
qPCR: Quantitative polymerase chain reaction
RA: Rhizoma Alismatis, the dried rhizome of *A. orientale*
SDS: Sodium dodecyl sulfate

## References

[1] Rogero MM, Calder PC (2018). Obesity, inflammation, toll-like receptor 4 and fatty acids. Nutrients..

[2] Pfeifer A, Hoffmann LS (2015). Brown, beige, and white: the new color code of fat and its pharmacological implications. Annu Rev Pharmacol Toxicol.

[3] Arner P, Langin D (2014). Lipolysis in lipid turnover, cancer cachexia, and obesity-induced insulin resistance. Trends Endocrinol Metab.

[4] Jernås M, Palming J, Sjöholm K, Jennische E, Svensson PA, Gabrielsson BG, Levin M, Sjögren A, Rudemo M, Lystig TC, Carlsson B, Carlsson LMS, Lonn M, Jernås M, Palming J, Sjöholm K, Jennische E, Svensson PA, Gabrielsson BG, Levin M, Sjögren A, Rudemo M, Lystig TC, Carlsson B, Carlsson LMS, Lonn M (2006). Separation of human adipocytes by size: hypertrophic fat cells display distinct gene expression. FASEB J.

[5] Onal G, Kutlu O, Gozuacik D, Dokmeci ES (2017). Lipid droplets in health and disease. Lipids Health Dis.

[6] Jaworski K, Sarkadi-Nagy E, Duncan RE, Ahmadian M, Sul HS (2007). Regulation of triglyceride metabolism. IV. Hormonal regulation of lipolysis in adipose tissue. Am J Physiol Gastrointest Liver Physiol.

[7] Ahmadian M, Duncan RE, Sul HS (2009). The skinny on fat: lipolysis and fatty acid utilization in adipocytes. Trends Endocrinol Metab.

[8] Frühbeck G, Méndez-Giménez L, Fernández-Formoso JA, Fernández S, Rodríguez A (2014). Regulation of adipocyte lipolysis. Nutr Res Rev.

[9] Zechner R, Zimmermann R, Kohlwein SD, Haemmerle G, Lass A, Eichmann TO (2012). Fat signals-lipases and lipolysis in lipid metabolism and signaling. Cell Metab.

[10] Sztalryd C, Brasaemle DL (1862). The perilipin family of lipid droplet proteins: gatekeepers of intracellular lipolysis. Biochim Biophys Acta Mol Cell Biol Lipids.

[11] Souza SC, Palmer HJ, Kang YH, Yamamoto MT, Muliro KV, Paulson KE (2003). TNF-alpha induction of lipolysis is mediated through activation of the extracellular signal related kinase pathway in 3T3-L1 adipocytes. J Cell Biochem.

[12] Tian T, Chen H, Zhao YY (2014). Traditional uses, phytochemistry, pharmacology, toxicology and quality control of *Alisma orientale* (Sam.) Juzep: a review. J Ethnopharmacol.

[13] Zhang LL, Xu W, Xu YL, Chen X, Huang M, Lu JJ (2017). Therapeutic potential of Rhizoma Alismatis: a review on ethnomedicinal application, phytochemistry, pharmacology, and toxicology. Ann N Y Acad Sci.

[14] Choi E, Jang E, Lee JH (2019). Pharmacological activities of *Alisma orientale* against nonalcoholic fatty liver disease and metabolic syndrome: literature review. Evid Based Complement Alternat Med.

[15] Nakajima Y, Satoh Y, Katsumata M, Tsujiyama K, Ida Y, Shoji J (1994). Terpenoids of *Alisma orientale* rhizome and the crude drug Alismatis Rhizoma. Phytochemistry..

[16] Xu F, Yu H, Lu C, Chen J, Gu W (2016). The cholesterol-lowering effect of alisol acetates based on HMG-CoA reductase and its molecular mechanism. Evid Based Complement Alternat Med.

[17] Xu F, Yu H, Lu C, Wu QN, Gu W, Chen J (2016). Study on alisols hypolipidemic effect and molecular mechanism. J Nanjing Univ Tradit Chin Med.

[18] Zeng L, Tang W, Yin J, Feng L, Li Y, Yao X, Zhou BJ (2016). Alisol A 24-acetate prevents hepatic steatosis and metabolic disorders in HepG2 cells. Cell Physiol Biochem.

[19] Wang Q, Wang ST, Yang X, You PP, Zhang W (2015). Myricetin suppresses differentiation of 3T3-L1 preadipocytes and enhances lipolysis in adipocytes. Nutr Res.

[20] Kraemer FB, Shen WJ (2002). Hormone-sensitive lipase: control of intracellular tri-(di-)acylglycerol and cholesteryl ester hydrolysis. J Lipid Res.

[21] Lindquist JM, Fredriksson JM, Rehnmark S, Cannon B, Nedergaard J (2000). Beta 3- and alpha 1-adrenergic Erk1/2 activation is Src- but not Gi-mediated in brown adipocytes. J Biol Chem.

[22] Liu LR, Lin SP, Chen CC, Chen YJ, Tai CC, Chang SC, Juang RH, Tseng YW, Liu BH, Mersmann HJ, Shen TL, Ding ST (2011). Serum amyloid a induces lipolysis by downregulating perilipin through ERK1/2 and PKA signaling pathways. Obesity (Silver Spring).

[23] Caimari A, Oliver P, Palou A (2012). Adipose triglyceride lipase expression and fasting regulation are differently affected by cold exposure in adipose tissues of lean and obese Zucker rats. J Nutr Biochem.

[24] Zechner R, Madeo F, Kratky D (2017). Cytosolic lipolysis and lipophagy: two sides of the same coin. Nat Rev Mol Cell Biol.

[25] Holm C (2003). Molecular mechanisms regulating hormone-sensitive lipase and lipolysis. Biochem Soc Trans.

[26] Drira R, Sakamoto K (2014). Hydroxytyrosol stimulates lipolysis via A-kinase and extracellular signal-regulated kinase activation in 3T3-L1 adipocytes. Eur J Nutr.

[27] Greenberg AS, Shen WJ, Muliro K, Patel S, Souza SC, Roth RA, Kraemer FB (2001). Stimulation of lipolysis and hormone-sensitive lipase via the extracellular signal-regulated kinase pathway. J Biol Chem.

[28] Arimura N, Horiba T, Imagawa M, Shimizu M, Sato R (2004). The peroxisome proliferator-activated receptor gamma regulates expression of the perilipin gene in adipocytes. J Biol Chem.

[29] Nagai S, Shimizu C, Umetsu M, Taniguchi S, Endo M, Miyoshi H, Yoshioka N, Kubo M, Koike T (2004). Identification of a functional peroxisome proliferator-activated receptor responsive element within the murine perilipin gene. Endocrinology..

[30] Li J, Mihalcioiu M, Li L, Zakikhani M, Camirand A, Kremer R (2018). Vitamin D prevents lipid accumulation in murine muscle through regulation of PPARgamma and perilipin-2 expression. J Steroid Biochem Mol Biol.

[31] Cingolani F, Czaja MJ (2016). Regulation and functions of autophagic lipolysis. Trends Endocrinol Metab.

[32] Wang C, Feng L, Ma L, Chen H, Tan X, Hou X (2017). Alisol A 24-acetate and alisol B 23-acetate induced autophagy mediates apoptosis and nephrotoxicity in human renal proximal tubular cells. Front Pharmacol.

[33] Wu C, Jing M, Yang L, Jin L, Ding Y, Lu J, Cao Q, Jiang Y (2018). Alisol A 24-acetate ameliorates nonalcoholic steatohepatitis by inhibiting oxidative stress and stimulating autophagy through the AMPK/mTOR pathway. Chem Biol Interact.

[34] Ahmadian M, Duncan RE, Varady KA, Frasson D, Hellerstein MK, Birkenfeld AL, Samuel VT, Shulman GI, Wang Y, Kang C, Sul HS (2009). Adipose overexpression of desnutrin promotes fatty acid use and attenuates diet-induced obesity. Diabetes..

[35] Caspar-Bauguil S, Kolditz CI, Lefort C, Vila I, Mouisel E, Beuzelin D, Tavernier G, Marques MA, Zakaroff-Girard A, Pecher C, Houssier M, Mir L, Nicolas S, Moro C, Langin D (2015). Fatty acids from fat cell lipolysis do not activate an inflammatory response but are stored as triacylglycerols in adipose tissue macrophages. Diabetologia..

[36] Langin D (2006). Adipose tissue lipolysis as a metabolic pathway to define pharmacological strategies against obesity and the metabolic syndrome. Pharmacol Res.

