# Supplementary figures and images for: Alisol A 24-acetate stimulates lipolysis in 3 T3-L1 adipocytes
Source: BMC Complement Med Ther. 2021 Apr 22;21:128. doi: 10.1186/s12906-021-03296-0 (PMC8063434; doi:10.1186/s12906-021-03296-0)

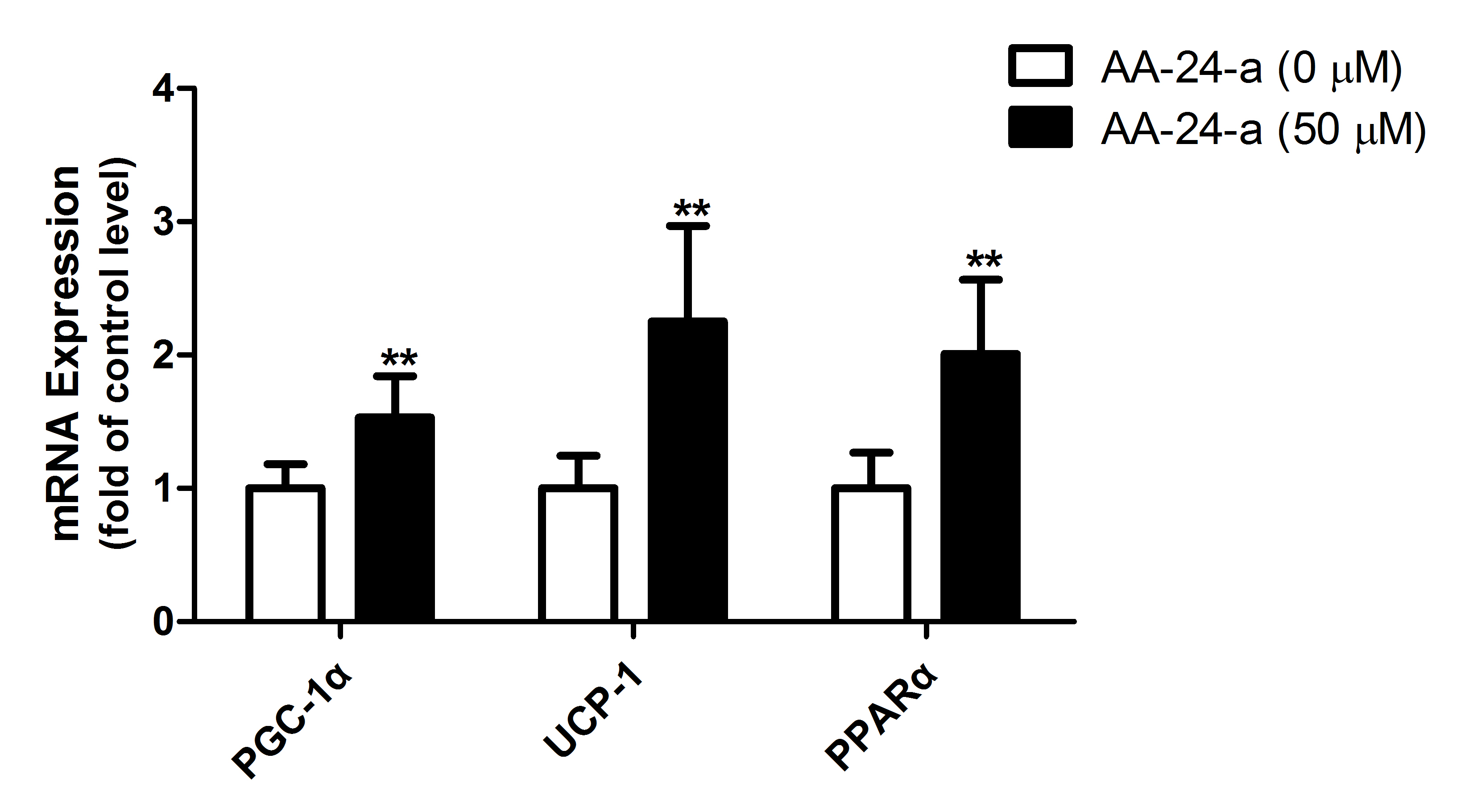

Supplement: Supplementary file 1 — Additional file 1: Supplementary Fig. S1. Relative mRNA expression levels of fatty acid oxidation-related genes in 3 T3-L1 adipocytes in response to treatment with AA-24-a for 12 h as measured by qPCR. Data are presented as mean ± standard deviation (n = 6). **P < 0.01, versus control cells. PGC-1α: PPARγ coactivator-1α; UCP-1: uncoupling protein-1. [file 12906_2021_3296_MOESM1_ESM.jpg]

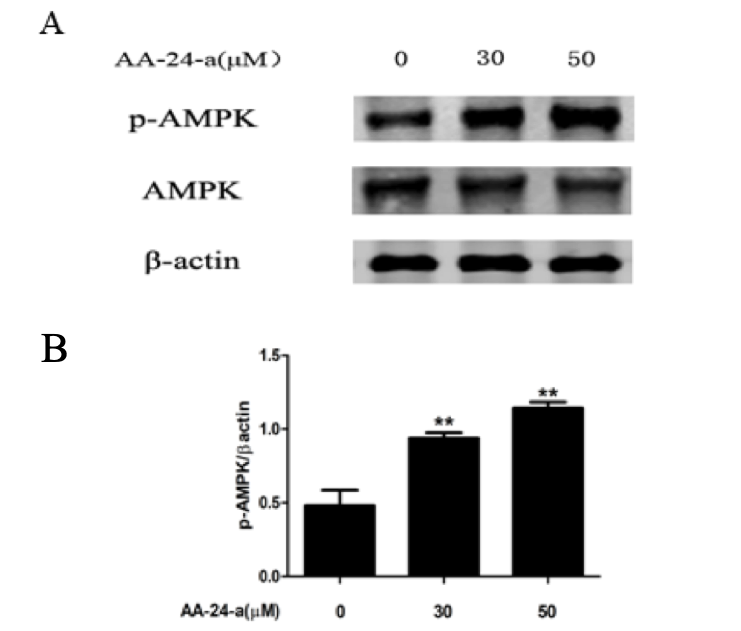

Supplement: Supplementary file 2 — Additional file 2: Supplementary Fig. S2. Effects of AA-24-a on AMPK and phospho-AMPK in 3 T3-L1 adipocytes. (A) Western blot analysis of protein expression after treatment with 0, 30, or 50 μM AA-24-a for 36 h. (B) Quantification of protein band densities as relative values against total β-actin. Data are presented as mean ± standard deviation (n = 3 or 4). *P < 0.05, **P < 0.01, versus control cells. [file 12906_2021_3296_MOESM2_ESM.png]

## Full blots of proteins

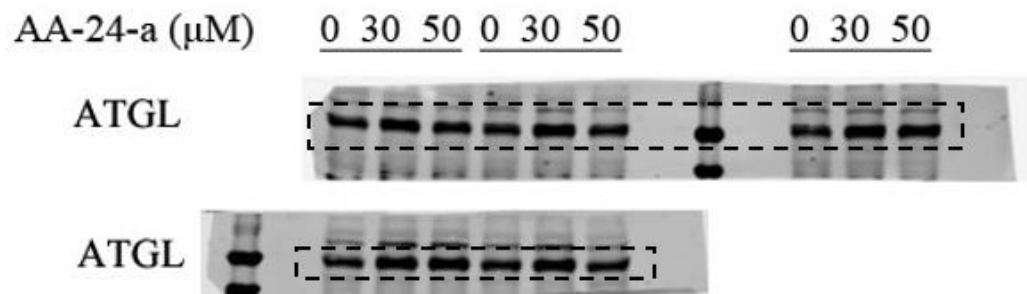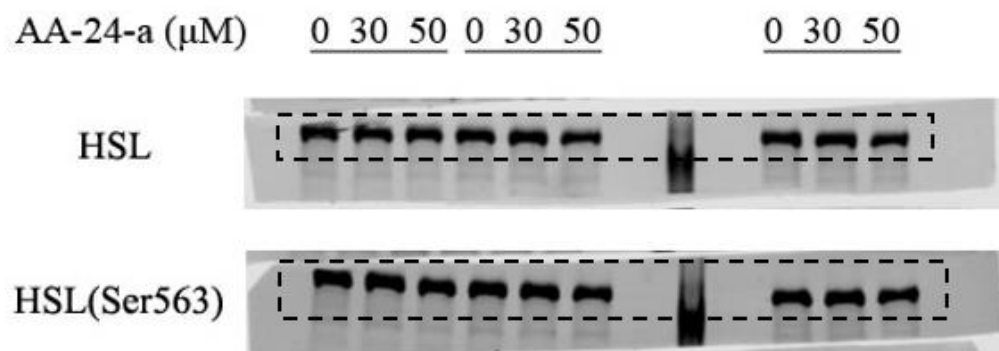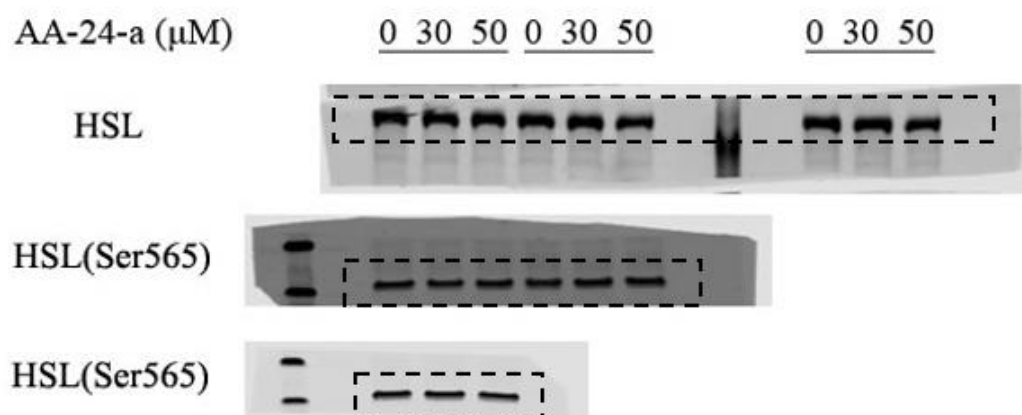

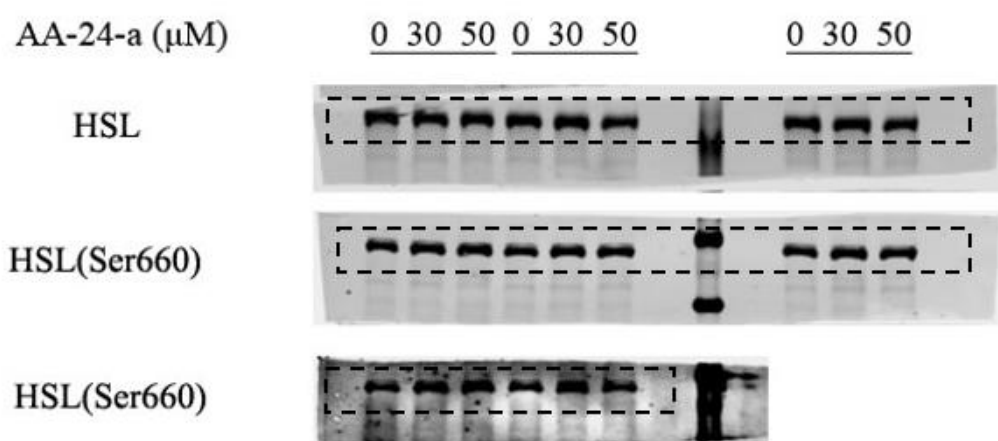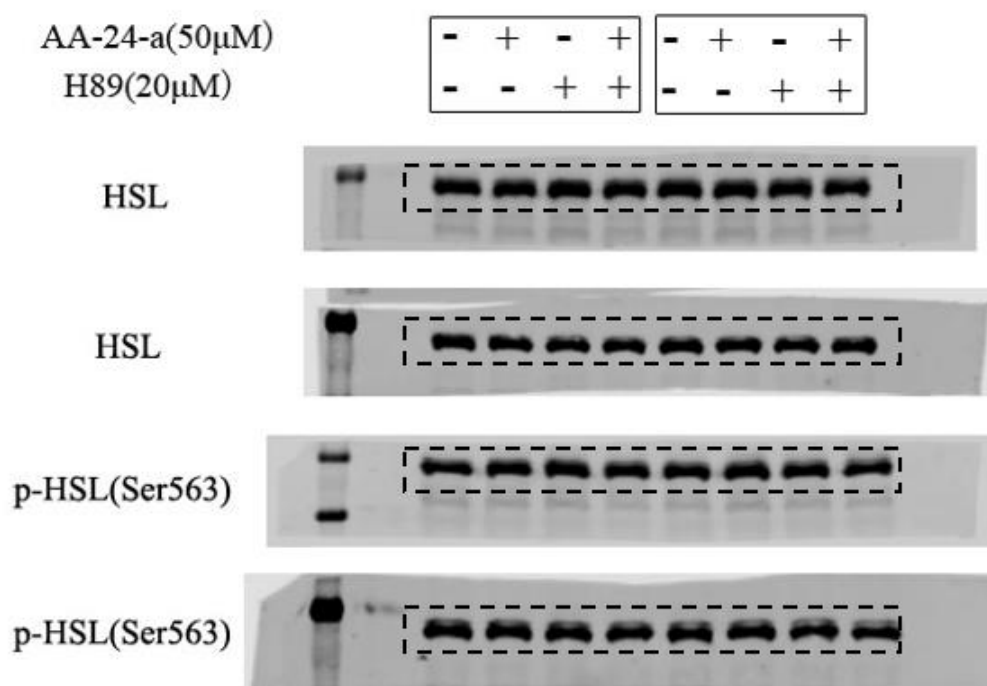

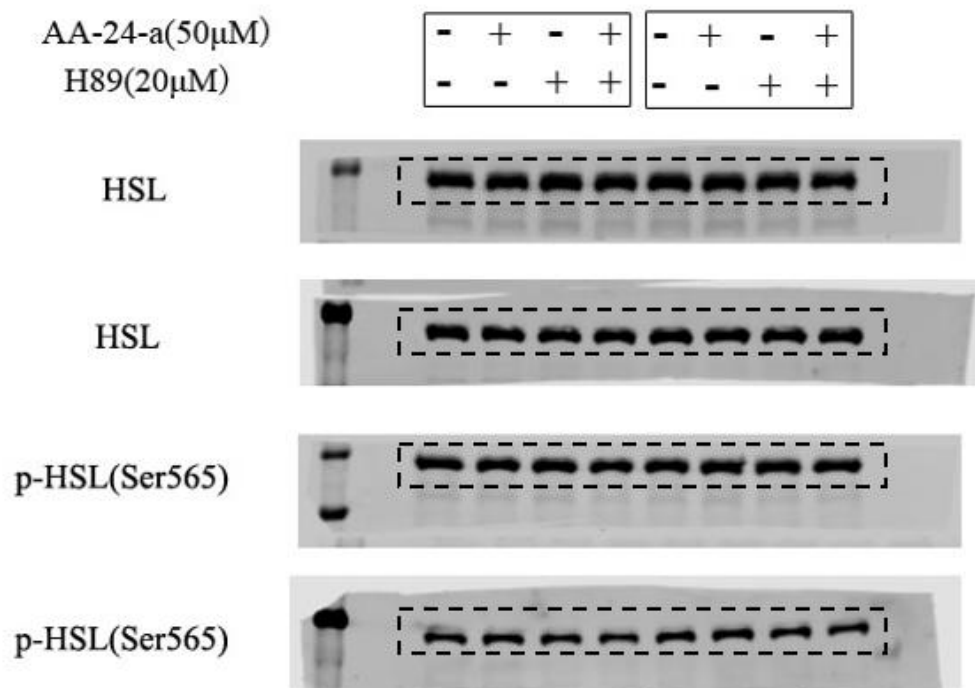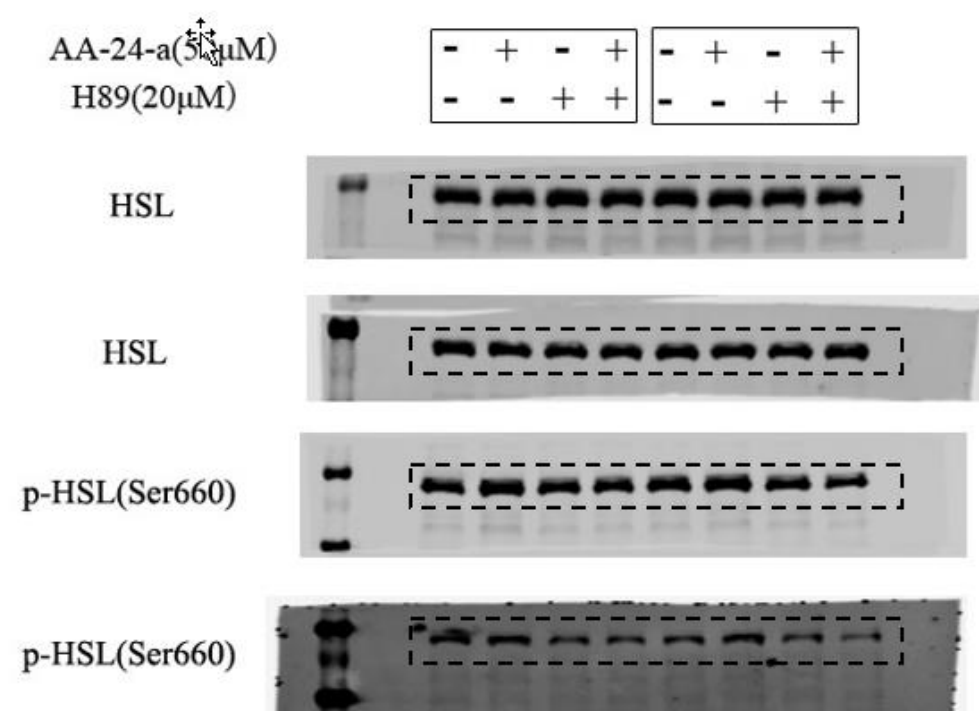

AA-24-a(50μM)

PD98059(50μM)

|   |   |   |   |   |   |   |   |
|---|---|---|---|---|---|---|---|
| - | + | - | + | - | + | - | + |
| - | - | + | + | - | - | + | + |

p-ERK

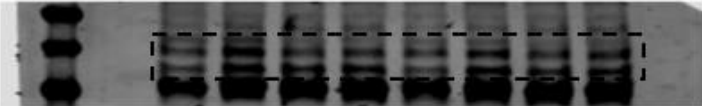

p-ERK

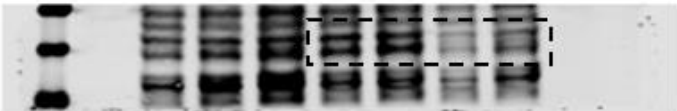

AA-24-a(50μM)

PD98059(50μM)

|   |   |   |   |   |   |   |   |
|---|---|---|---|---|---|---|---|
| - | + | - | + | - | + | - | + |
| - | - | + | + | - | - | + | + |

PPAR $\gamma$

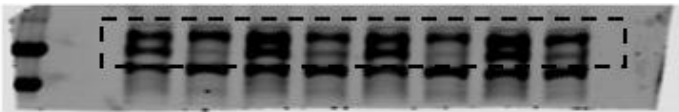

PPAR $\gamma$

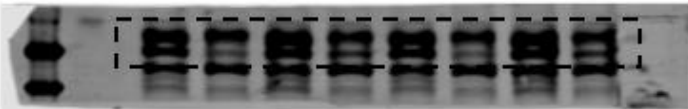

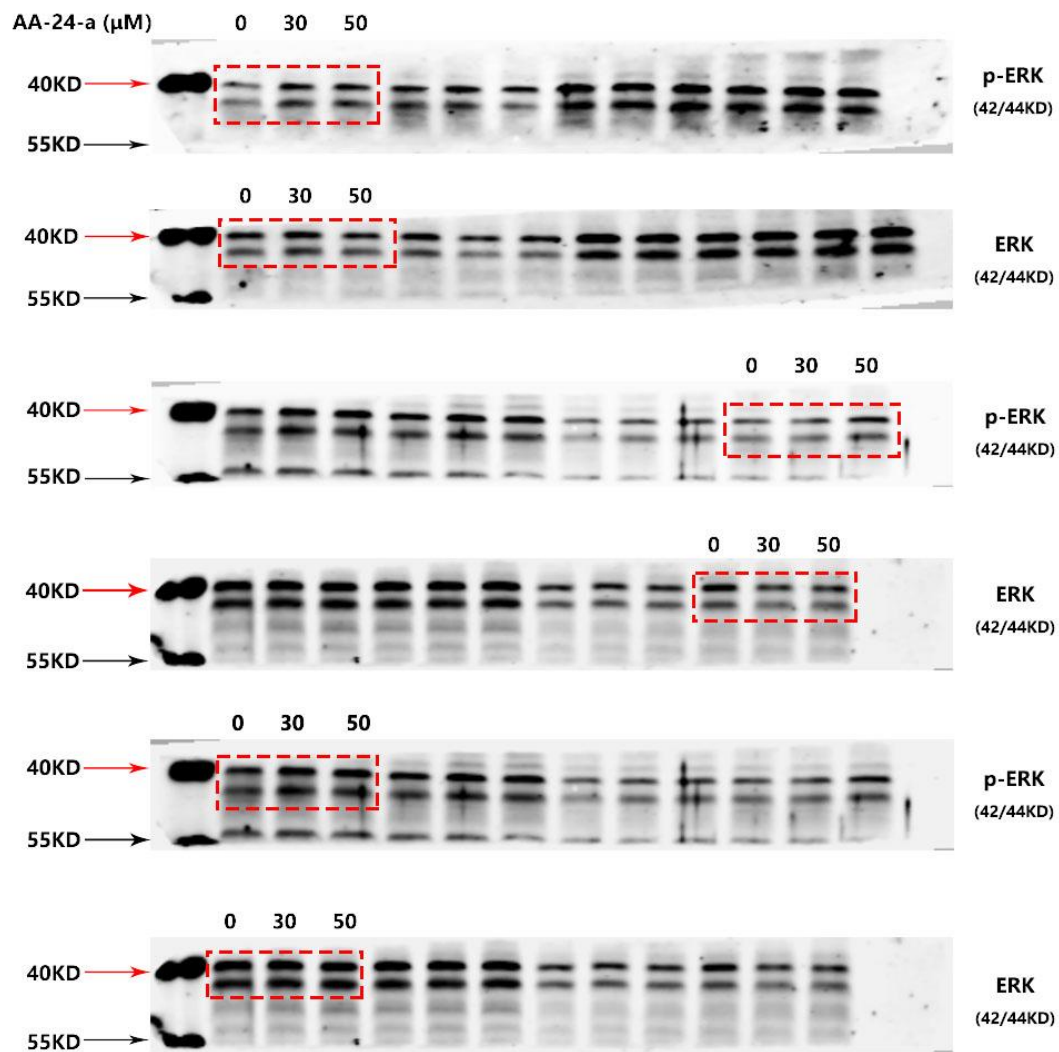

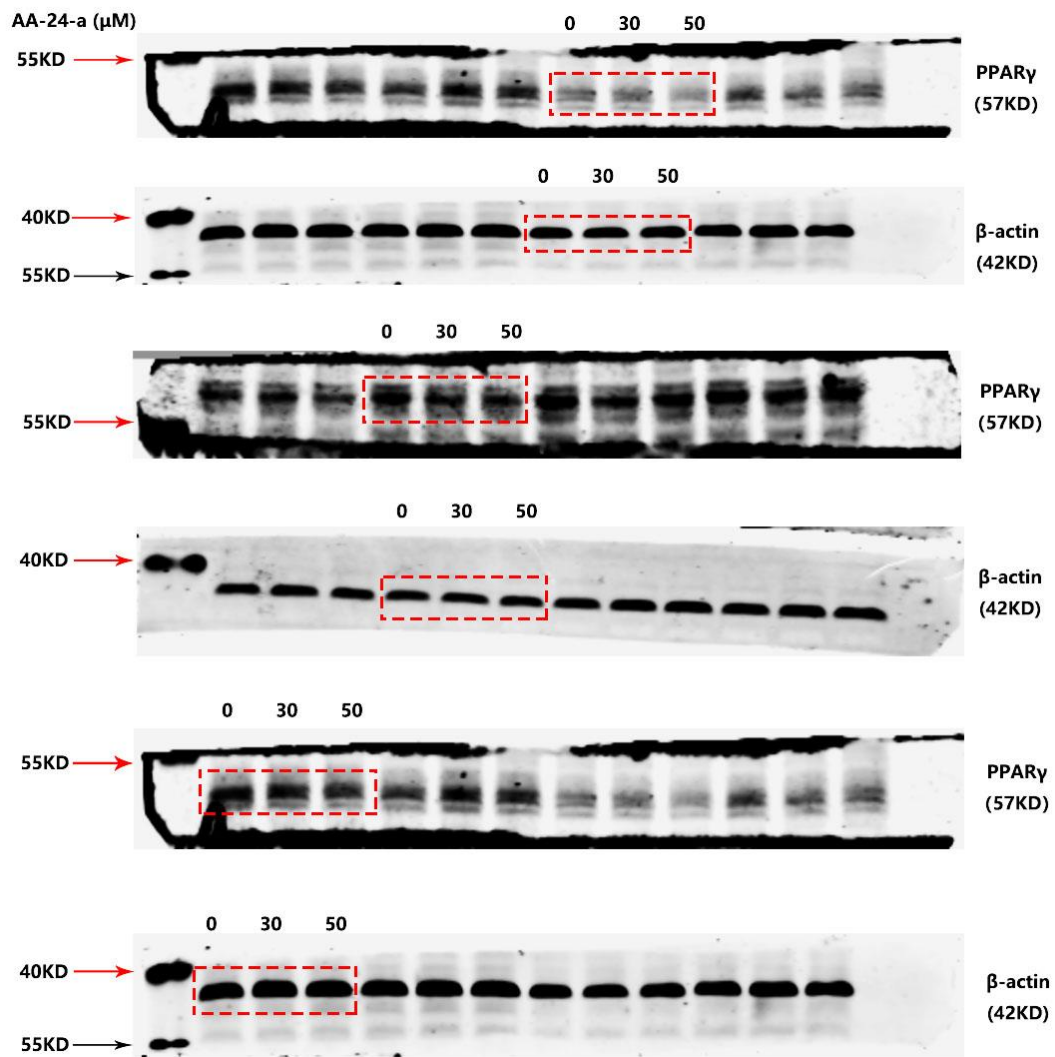

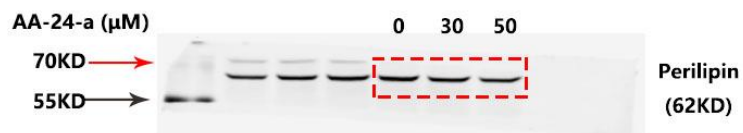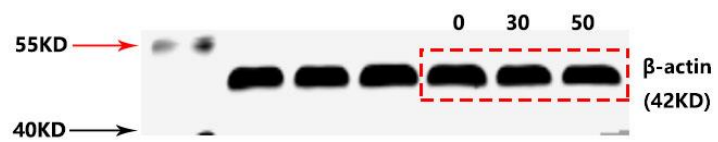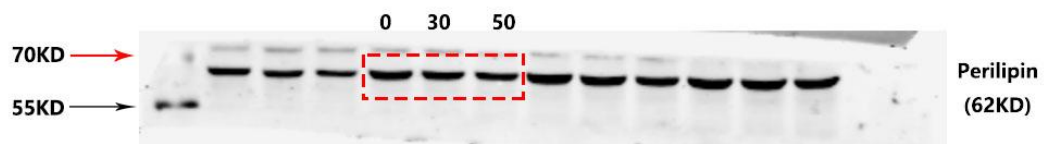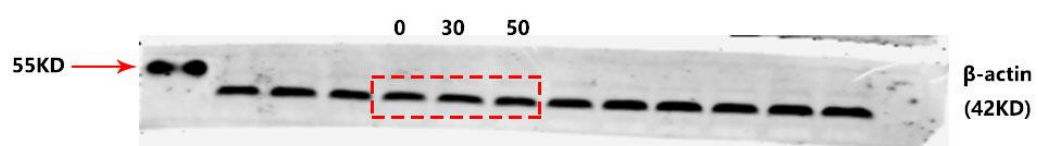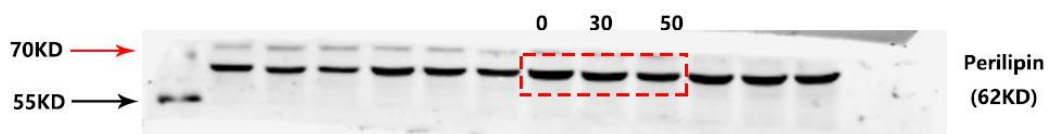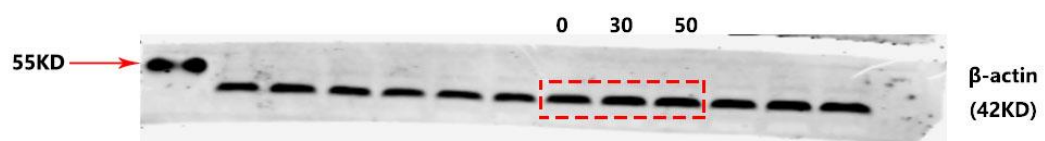

Supplement: Supplementary file 3 — Additional file 3: Supplementary Material. Full western blots of proteins. [file 12906_2021_3296_MOESM3_ESM.pdf]
